# Examining the differential protective effects of women’s spirituality and religiosity on alcohol and marijuana use by sexual identity

**DOI:** 10.1016/j.abrep.2022.100450

**Published:** 2022-08-13

**Authors:** Laurie A. Drabble, Amy A. Mericle, Cat Munroe, Alison Cerezo, Katherine J. Karriker-Jaffe, Tonda L. Hughes, Karen F. Trocki

**Affiliations:** a San José State University College of Health and Human Sciences, One Washington Square, San Jose, CA 95192-0049, USA; b Alcohol Research Group, Public Health Institute, USA; c Department of Counseling, Clinical & School Psychology, University of California Santa Barbara, USA; d RTI, International, USA; e School of Nursing & Department of Psychiatry, Columbia University, USA

**Keywords:** Sexual minority women, Religiosity, Spirituality, Alcohol, Alcohol use disorder, Marijuana

## Abstract

The current study explored how religiosity and spirituality may differentially influence substance use by sexual identity based on a sample of adult sexual minority women (SMW; n = 437 lesbian; n = 323 bisexual) relative to a heterosexual comparison sample (n = 636). We examined three questions: (1) whether *spirituality* was differentially associated with alcohol and marijuana use by sexual identity; (2) whether *religiosity* was differentially associated with alcohol and marijuana use by sexual identity; (3) whether observed differences between spirituality or religiosity and substance use by sexual identity persisted after adjusting for religious environment. Measures included spirituality (importance of spirituality), religiosity (importance of religion, attending religious services), and past year substance use (alcohol use disorder [AUD], any marijuana use, and regular marijuana use). Higher levels of spirituality were associated with increased odds of AUD among both lesbian and bisexual respondents relative to heterosexuals. Higher levels of religiosity among lesbian participants were associated with increased odds of AUD relative to heterosexuals with higher levels of religiosity. Consistent with theories of minority stress, findings suggest that spirituality and religiosity are less protective for SMW than heterosexual women and, in some cases, may contribute to greater risk of substance use.

## Highlights

• Religiosity and spirituality impact substance use differently by sexual identity.
• Religiosity and spirituality were protective against substance use in heterosexuals.
• Religiosity was not protective against alcohol use disorders (AUD) among lesbians.
• Greater spirituality predicted higher odds of AUD among lesbians and bisexuals.

## 1 Introduction

Religion and spirituality play complex roles in the health of sexual minorities. For example, they may support positive coping with challenging life circumstances. However, many major religious traditions are non-affirming of same sex attractions and behaviors (Whitley, 2009), thereby contributing to stigma and oppression that undermine the potential health and psychological benefits often associated with religion and spirituality. For example, one U.S. study found that exposure to religious prejudice was associated with negative health outcomes among sexual minorities, including higher levels of stress, anxiety, shame, harmful alcohol use, and more instances of experiencing physical and verbal abuse (Sowe et al., 2017). Similarly, findings from systematic reviews and *meta*-analyses suggest that while some sexual minorities find social support and refuge in religious traditions, others report religious affiliation and religion as a source of stigma and stress (Lefevor et al., 2021, Rodriguez, 2009, Wilkinson and Johnson, 2020).

Although religiosity has been found to be protective against hazardous alcohol and drug use in the general population (Allen & Lo, 2010), findings regarding this relationship are mixed in studies with sexual minorities (Lefevor et al., 2021). Understanding factors that may protect against hazardous alcohol and marijuana use is important in the context of persistent sexual identity-related disparities in substance use (Drabble et al., 2020, Fish and Baams, 2018, McCabe et al., 2021). Examining factors that may affect alcohol and marijuana use among sexual minority women (SMW; e.g., lesbian, bisexual, and queer identified women) is particularly important given research documenting higher rates of hazardous drinking and marijuana use among SMW compared to heterosexual women, and disparities by sexual identity that are generally more pronounced among women than among men (Drabble et al., 2005, Drabble et al., 2020, Hughes et al., 2020, Hughes et al., 2016, McCabe et al., 2009).

### 1.1 Religiosity and spirituality among sexual minorities

Religiosity and spirituality constitute separate yet related phenomena. Religiosity represents involvement in the rituals, cultural traditions and practices of a particular religious institution or community (Aldwin et al., 2014). Spirituality represents an individual’s beliefs and practices related to a higher power, search for meaning, or sense of transcendence, which may be secular or linked with religion (Aldwin et al., 2014, Allen and Lo, 2010). Research suggests that sexual minorities generally consider spirituality as having greater importance in their life than religion (Drabble, Veldhuis, et al., 2018; Halkitis et al., 2009). Relative to heterosexuals, sexual minorities are less likely to attend religious services or to consider religion as somewhat or very important in their lives (Murphy, 2015, Schwadel and Sandstrom, 2019), however these differences are smaller in relation to measures of spirituality (Schwadel & Sandstrom, 2019).

Although general population studies have found small to medium positive health effects for religion and spirituality, using *meta*-analysis, Lefevor and colleagues (2021) found much smaller positive relationships with health outcomes among sexual minorities. Findings were also inconsistent among sexual minorities and relationships varied depending on how religion and spirituality were measured. Measures of spirituality were positively associated with health, but measures of religious attendance were not (Lefevor et al., 2021). The absence of a positive relationship between religious attendance and health among sexual minorities may be partially explained by exposure to unique stigma-related stressors in religious contexts (Wilkinson & Johnson, 2020). Research with general population samples has found strong associations between higher religiosity (e.g., frequency of attendance at religious services; self-rated religiosity) and negative attitudes toward sexual minorities (Whitley, 2009). Furthermore, close to one-third of sexual minority adults in a U.S. survey reported feeling unwelcome in a place of worship (Pew Research, 2013). Exposure to religious heterosexist stigma is, in turn, associated with negative health, mental health, and substance use outcomes among sexual minorities (Sowe et al., 2017, Wilkinson and Johnson, 2020). This complexity of relationships between religiosity and spirituality (Lefevor et al., 2021) underscores the importance of multiple measures of religiosity and spirituality in research with sexual minority adults.

### 1.2 Religion, spirituality and substance use among sexual minority women

Literature on the relationship between religion and/or spirituality and alcohol or marijuana use among SMW is limited and shows mixed results (Hughes et al., 2020, Lefevor et al., 2021). One study with SMW found that neither religiosity nor spirituality predicted past-year substance use outcomes of hazardous drinking (i.e., dependence symptoms, heavy episodic drinking, intoxication, adverse alcohol-related consequences) or drug use, including marijuana (Drabble, Veldhuis, et al., 2018). Another study found that religiosity was protective against hazardous drinking and drug use among both SMW and heterosexual women (Drabble et al., 2016). Associations between religiosity and alcohol use may also differ between bisexual and lesbian women (Schulz et al., 2021). One study found that religiosity was protective against heavy episodic drinking among heterosexual women; however, it was not protective for lesbian women and it was associated with *increased* drinking among bisexual women (Rostosky et al., 2014). The authors hypothesize that relative to religious lesbian women, religious bisexual women may have less social support in lesbian and gay communities to counteract potential stigmatizing experiences. They may also have fewer role models for positive bisexual identity and experience greater pressure to adhere to heterosexist scripts. In the context of these mixed findings, research exploring the relationships between religion and spirituality and substance use outcomes, and in particular disaggregating findings for lesbian and bisexual women, is warranted.

### 1.3 The current study

This study used data from SMW recruited from two web-based panels and from a sample of heterosexual women participating in a nationally representative study of alcohol use. We tested: (1) whether *spirituality* was differentially associated with alcohol and marijuana use by sexual identity; (2) whether *religiosity* was differentially associated with alcohol and marijuana use by sexual identity; (3) whether observed differences between spirituality or religion and substance use by sexual identity persisted after adjusting for religious environment, defined as the degree to which women reported affiliation with churches that were welcoming of lesbian, gay, bisexual and transgender (LGBT) individuals.

## 2 Methods

### 2.1 Participants

SMW participants were recruited from two national online panels: a general population panel and an LGBT-specific panel. Eligibility for participation in the panel samples was restricted to participants ages 18 or older and who identified as lesbian, bisexual, or other non-exclusively heterosexual identity; resided in the U.S.; and identified as women at the time of the screening. The LGBT-specific panel (CMI) was drawn from a diverse panel of over 50,000 LGBT participants across all states in the U.S., including 20,000 SMW, who were originally recruited through partnerships with over 300 LGBT websites, publications, organizations, apps and social media. The general population panel (MFour) included approximately 2.5 million active participants in the U.S, recruited using a wide range of methods to obtain a geographically and demographically diverse sample of participants over age 13, in all 50 states, who own a smartphone and are registered to receive and respond to survey opportunities through an app. To over-sample SMW who identified as African American or Latinx, each wave of recruitment targeted a random sample that was one-third African American/Black, one-third Latinx, and one-third unrestricted by race/ethnicity. Participants were compensated through the panel companies following their standard payment protocols. The participation rate for the general panel sample (adjusted for eligibility) was 45 % and the participation rate for the LGBT sample was 28.7 %.

Heterosexual participants were recruited from a pool of former participants in the National Alcohol Survey (NAS), a national probability survey. The NAS is a cross-sectional probability survey of adults ages 18 or older in the U.S., conducted approximately every-five years that used computer-assisted interviewer (CATI) with a random sample of both landlines and cell phones with oversampling in low-population states and oversampling in Black- and Latinx-dense areas. Participants from the probability survey were eligible for random selection in the present study if they selected “female” as their gender and “only heterosexual or straight” in response to a question asking them to choose the category that best described their sexual orientation. A random sample of 1,961 heterosexual women who participated in the 2015 NAS were invited to participate in the current study. Computer assisted telephone interviews were completed with 623 respondents (40.56 % response rate).

The general panel sample and the national probability survey sample included only binary “male/female” response options and did not assess whether respondents were assigned female at birth. The LGBT-specific panel allowed participants to select multiple sex and gender identities; however, to be consistent with categorizations in the general panel and probability samples, only participants from this panel were included in the current study if they selected “female” as their gender (even if they also selected other identities). Although we refer to participants as “women” in this paper, we acknowledge that study participants may have endorsed other gender categories had they been provided such options. The SMW samples were initially screened based on endorsing sexual minority identity and the heterosexual comparison sample was selected based on prior endorsement of heterosexual identity; the few participants who selected “mostly heterosexual” in the current study were combined with those endorsing heterosexual or straight identity.

As shown in Table 1, 44.6 % of the study sample was from the national population-based survey, one-quarter (25.1 %) was from the LGBT panel sample, and close to one-third (30.2 %) was from the general panel sample. Approximately 46 % of the sample identified as heterosexual; 23 % identified as bisexual and 31 % as lesbian. The majority of the sample was<50 years old (63 %), college-educated (77 %), currently employed (62 %), and currently partnered (65 %); just under one-half identified their race as White. Although the majority also reported being Protestant, Catholic, Jewish, or some other religion, a quarter of the sample (25 %) reported not having a religious affiliation. Table 1 displays characteristics by sexual identity. Differences by sexual identity were found with respect to age, race/ethnicity, educational attainment, current employment, partner status, current religion, current religious environment, and study sample.

**Table 1:** Unweighted Sample Characteristics by Sexual Identity (N = 1,396).

		Full Sample (N = 1,396)	Heterosexual (N = 636)		Bisexual (N = 323)	Lesbian (N = 437)	
		*n (*%)	*n* (%)		*N* (%)	*N* (%)	
Age (N = 1,383)							***
	18–29	394 (28.5)	78 (12.5)		158 (49.4)	158 (36.2)	
	30–49	475 (34.4)	161 (25.7)		136 (42.5)	178 (40.7)	
	50+	514 (37.2)	387 (61.8)		26 (8.1)	101 (23.1)	
Race/Ethnicity							***
	White	638 (45.7)	354 (55.7)		132 (40.9)	152 (34.8)	
	Black	371 (26.6)	176 (27.7)		71 (22.0)	124 (28.4)	
	Latinx	317 (22.7)	78 (12.3)		101 (31.3)	138 (31.6)	
	Other/Missing	70 (5.0)	28 (4.4)		19 (5.9)	23 (5.3)	
Educational Attainment (n = 1,394)							***
	<High school	63 (4.5)	44 (6.9)		13 (4.0)	6 (1.4)	
	High school	257 (18.4)	137 (21.6)		64 (19.8)	56 (12.8)	
	Some college	455 (32.6)	183 (28.9)		130 (40.3)	142 (32.5)	
	College+	619 (44.4)	270 (42.6)		116 (35.9)	233 (53.3)	
Currently Employed		868 (62.2)	295 (46.4)		223 (69.0)	350 (80.1)	***
Partnered (N = 1,395)		912 (65.4)	392 (61.7)		220 (68.1)	300 (68.7)	*
Current Religion							***
	Protestant	419 (30.0)	268 (42.1)		49 (15.2)	102 (23.3)	
	Catholic	299 (21.4)	116 (18.2)		73 (22.6)	110 (25.2)	
	Jewish	47 (3.4)	18 (2.8)		15 (4.6)	14 (3.2)	
	Something else	282 (20.2)	136 (21.4)		68 (21.1)	78 (17.9)	
	No religious affiliation/missing	349 (25.0)	98 (15.4)		118 (36.5)	133 (30.4)	
Religious Environment (N = 1,305)							***
	Not a member	899 (68.9)	266 (48.5)		265 (82.8)	368 (84.4)	
	Welcoming of LGBT people	154 (11.8)	57 (10.4)		41 (12.8)	56 (12.8)	
	Unwelcoming of LGBT people	252 (19.3)	226 (41.2)		14 (4.4)	12 (2.8)	
Sample							***
	LGBT-specific panel	351 (25.1)	2 (0.3)		98 (30.3)	251 (57.4)	
	General population panel	422 (30.2)	16 (2.5)		220 (68.1)	186 (42.6)	
	Heterosexual recontact	623 (44.6)	618 (97.2)		5 (1.6)	0 (0.0)	
Spirituality and Religiosity		M (SD)	M (SD)		M (SD)	M (SD)	
	Spirituality Score (1–4)	3.18 (1.01)	3.54	0.79	2.84 (1.08)	2.92 (1.09)	***
	Religiosity (1–4)	2.76 (1.19)	3.40	0.92	2.26 (1.11)	2.19 (1.14)	***
	Religious attendance (1–5)	2.71 (1.55)	3.57	1.49	2.05 (1.27)	1.95 (1.11)	***

*Notes*. Valid percentages are listed; missing data was minimal. Differences by sexual identity were tested with Chi-square and Fisher's Exact tests.

* *p* < 0.05, **p < 0.01.

*** *p* < 0.001.

### 2.2 Procedures

Panel sample participants were invited in 2019 to complete an online survey that included a range of questions related to substance use and factors known to be predictive of hazardous drinking and drug use. Heterosexual women who previously participated in the NAS were again recruited in 2016 to complete a supplemental (CATI) survey. The goal of recontacting heterosexual women participants in the NAS was to administer measures that were included in the panel surveys but were not asked in the original NAS survey. Data from these sources were merged for analysis in the current study. All procedures were reviewed and approved by the institutional IRB.

### 2.3 Measures

#### 2.3.1 Sexual identity

Sexual identity was determined based on the question, “Recognizing that sexual identity is only part of your identity, which of the following statements best describes your sexual orientation?” Respondents were provided the following options: Only heterosexual; Mostly heterosexual; Bisexual; Mostly lesbian or gay; Only lesbian or gay; Something else (McCabe et al., 2012). We constructed a three-category variable from the responses: heterosexual (including mostly heterosexual), bisexual (including participants who endorsed pansexual, fluid or other non-monosexual identity), and lesbian (including mostly lesbian).

#### 2.3.2 Demographic and other covariates

In our multivariable models, we adjusted for a number of demographic and other covariates. These included age (18–29, 30–49, or 50 + ), race/ethnicity (White, Black, Latinx, or other/missing), educational attainment (less than high school, high school, some college, or college or greater), current employment (yes/no), and whether individuals were currently in a “partnered” relationship (married, living with a partner in a committed relationship, or in a committed relationship but not living with a partner). We also adjusted for religious affiliation (Protestant, Catholic, Jewish, something else, and no religious affiliation/missing). Finally, we adjusted for the sample from which the participant was recruited.

#### 2.3.3. Spirituality

Spirituality was defined as how often respondents spent time thinking about the ultimate purpose of life or their own relationship to a higher power in life. Participants rated the importance of spirituality in their lives on a 4-point scale ranging from “not at all important” to “not very important” (Drabble, Veldhuis et al., 2018), coded such that higher scores represented greater importance of spirituality; M = 3.18, SD = 1.01.

#### 2.3.4 Religiosity

Religiosity reflected participants’ feelings and behavior. Participants rated the importance of religion in their lives on a 4-point scale ranging from “not at all important” to “very important” (Drabble et al., 2016, Michalak et al., 2007), coded such that higher scores represented greater religiosity; M = 2.76, SD = 1.19. Participants also indicated how often they attended religious services on a five-point Likert scale of never, rarely, a few times during the year, about once or twice a month, or once a week or more (Rostosky et al., 2014). Higher scores reflect greater religiosity; M = 2.71, SD = 1.55.

#### 2.3.5 Religious environment

Participants who endorsed attending religious services were asked about the environment where they attended services. Specifically, SMW respondents in the panel samples who attended religious services were asked whether the place they attend was welcoming of LGBT people. Respondents in the heterosexual resample were asked if their congregation had adopted a statement that officially welcomes gays and lesbians. A three-category variable was created for analysis: not a member of a religious organization, attended congregation welcoming of LGBT people, and attended congregation unwelcoming of LGBT people.

#### 2.3.6 Alcohol measure

We created a dichotomous indicator of whether participants met criteria for past year alcohol use disorder (AUD) as set forth in the 5th edition of the American Psychiatric Association’s Diagnostic and Statistical Manual (DSM-5; American Psychiatric Association, 2013). Participants were asked about symptoms in 11 domains (failure to fulfill role obligations; drinking despite social or interpersonal problems; drinking when physically hazardous; tolerance; withdrawal; using alcohol more than or for longer than intended; persistent desire to cut down/control use; giving up important activities; spending a lot of time getting alcohol, using alcohol or recovering from use; drinking despite physical or psychological problems; and craving). Participants who endorsed two or more criteria (mild to severe AUD) were classified as positive for AUD.

#### 2.3.7 Marijuana use measures

Participants were asked how often they used marijuana, hash, pot, THC, or ‘weed’ during the last twelve months. Response options included every day or nearly every day, about once a week, once every 2 or 3 weeks, once every month or two, less often than that, and never. Two dichotomous variables were constructed. Any use was constructed as any past year use vs none. Regular use was constructed as use once every month or two or more times a month vs less frequent or no use.

### 2.4 Statistical analyses

All analyses were conducted in Stata (version 16) using sample weights and variance estimation techniques that adjusted for the complex design features of the NAS recontact and panel surveys. We first conducted separate logistic regression analyses to test the independent effects of spirituality and religiosity measures and sexual identity on the alcohol and marijuana outcomes. Wald tests were performed to test the overall effect of variables with multiple categories. We then ran separate models including the interaction between the spirituality and religiosity measures with sexual identity to examine the differential effects of sexual identity on the relationship between spirituality and religiosity with the alcohol and marijuana outcomes. These models adjusted for the demographic and other covariates listed above. In these interaction models, contrasts tested the joint effects of the interaction. In addition to presenting model coefficients, we also graphically display predictive margins for models in which interactions were statistically significant. We then reran these models adding the covariate of religious environment to examine whether any observed differences between religion and substance use measures by sexual identity persisted.

## 3 Results

### 3.1 Independent effects models

Table 2 presents findings from weighted logistic regression analyses testing the independent effects of the spirituality and religiosity measures and of sexual identity on alcohol and marijuana use. Greater levels of importance of spirituality were associated with lower odds of meeting criteria for past year AUD (OR = 0.71, p = 0.037), and any marijuana use (OR = 0.76, p = 0.017). Greater levels of religiosity, *reflected by importance of religion* were associated with lower odds of meeting criteria for past year AUD (OR = 0.58, p < 0.001), any marijuana use (OR = 0.62, p < 0.001), and regular marijuana use (OR = 0.63, p < 0.001). Greater levels of religiosity, *reflected by religious attendance* were associated with lower odds of meeting criteria for past year AUD (OR = 0.71, p = 0.017), any marijuana use (OR = 0.57, p < 0.001), and regular marijuana use (OR = 0.59, p < 0.001).

**Table 2:** Independent Effects of Spirituality and Religiosity and Sexual Identity on Drinking and Marijuana Use (Weighted).

		Past Year AUD			Any Marijuana Use			Regular Marijuana Use		
		OR	SE	*p*	OR	SE	*p*	OR	SE	*p*
Spirituality		0.71	0.12	0.037	0.76	0.09	0.017	0.79	0.11	0.098
Religiosity		0.58	0.09	<0.001	0.62	0.07	<0.001	0.63	0.08	<0.001
Religious Attendance		0.71	0.10	0.017	0.57	0.05	<0.001	0.59	0.06	<0.001
Sexual Identity										
	Heterosexual (Ref)									
	Bisexual	4.87	2.16	<0.001	4.87	1.39	<0.001	4.18	1.31	<0.001
	Lesbian	1.70	0.57	0.112	6.85	1.67	<0.001	5.40	1.53	<0.001
	Wald Test			0.002*			<0.001			<0.001

*Notes.* Survey weighted logistic regression models tested the independent effects of spirituality and sexual identity on past year: meeting DSM5 AUD criteria, using any marijuana, and using marijuana at least every month or two. NAS weights capture the probability of being selected into the original NAS data, and do not account for nonresponse in the heterosexual recontact sample. The Wald Test represents the overall test of sexual identity on the outcome of interest. When the overall test was significant, post-hoc tests were run varying reference groups to test for differences between individuals in the bisexual category and those in the lesbian category.

* Bisexual significantly higher odds compared to lesbian respondents.

Sexual identity was significantly associated with all alcohol and marijuana outcomes. For the most part, participants identifying as sexual minorities were more likely than heterosexual women to report substance use outcomes, with no differences among sexual minority participants. The only exception was for past year AUD. For this outcome, bisexual women were more likely than heterosexual women to meet AUD criteria (OR = 4.87, p < 0.001), but there were no differences between lesbian and heterosexual women. Post-hoc tests varying the reference category in sexual identity variable revealed sexual minority group differences: bisexual women were nearly-three times as likely as lesbian women to meet criteria for AUD (OR = 2.82, p = 010; data not shown).

### 3.2 Interaction models

**Fig. 1:**
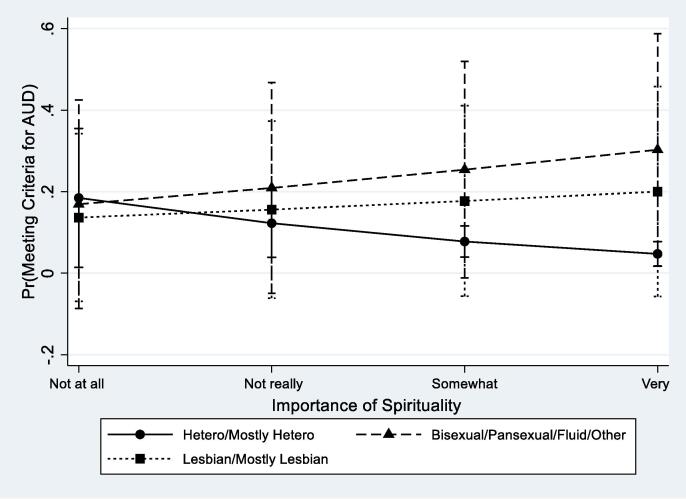
Predictive Margins for Alcohol Use Disorder (AUD) by Spirituality and Sexual Identity with 95% CIs.

**Fig. 2:**
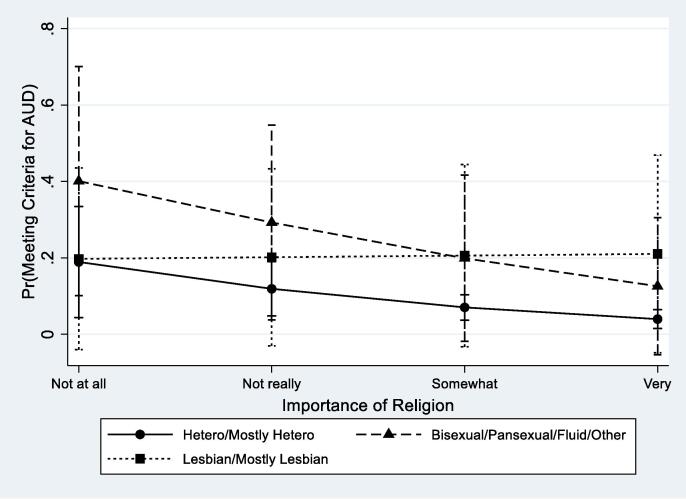
Predictive Margins for Alcohol Use Disorder (AUD) by Religious Importance and Sexual Identity with 95% CIs.

Table 3 displays findings from weighted logistic regression models testing the interactions between spirituality and religiosity and sexual identity on the study outcomes while adjusting for demographic covariates. Interactions were significant only for AUD. The Wald test of the interaction indicated differential effects of importance of spirituality by sexual identity (F = 3.50, p = 0.030) as well as importance of religion (F = 3.25, p = 0.039). Fig. 1, Fig. 2 graphically depict the nature of these interactive effects. Increases in importance of spirituality and importance of religion were associated with lower odds of meeting criteria for AUD among heterosexual women, but this was not the case for SMW. In fact, greater levels of importance of spirituality were associated with higher odds of AUD among both lesbian and bisexual women relative to heterosexual women (see Fig. 1). By contrast, the effect of religious importance was similar among heterosexual and bisexual participants, with higher religiosity associated with lower odds of AUD. However, we found no variation in odds of AUD by levels of religious importance among lesbian women (see Fig. 2).

**Table 3:** Models Testing the Interactions Between Measures of Spirituality and Religiosity with Sexual Identity on Substance Use Measures (Weighted).

		Past Year AUD			Any Marijuana Use			Regular Marijuana Use		
		aOR	SE	*p*	aOR	SE	*p*	aOR	SE	*p*
Spirituality		0.56	0.16	0.047	1.04	0.29	0.877	1.33	0.46	0.404
Sexual Identity										
	Heterosexual (Ref)									
	Bisexual	0.36	0.54	0.499	0.28	0.27	0.186	0.38	0.42	0.380
	Lesbian	0.30	0.42	0.386	0.11	0.11	0.030	0.19	0.22	0.152
Spirituality*Sexual Identity										
	Heterosexual (Ref)									
	Bisexual	2.45	0.89	0.014	1.03	0.31	0.917	0.75	0.27	0.427
	Lesbian	2.14	0.69	0.019	1.60	0.48	0.113	1.12	0.42	0.762
	Wald Test of the Interaction			**0.030**			0.102			0.259
Religiosity		0.52	0.13	0.010	0.87	0.20	0.550	1.03	0.28	0.909
Sexual Identity										
	Heterosexual (Ref)									
	Bisexual	4.10	4.56	0.204	0.27	0.22	0.108	0.31	0.26	0.162
	Lesbian	0.53	0.62	0.589	0.19	0.16	0.049	0.26	0.23	0.120
Religiosity*Sexual Identity										
	Heterosexual (Ref)									
	Bisexual	1.00	0.31	0.993	1.07	0.27	0.778	0.79	0.23	0.423
	Lesbian	2.01	0.62	0.025	1.45	0.35	0.125	1.04	0.29	0.875
	Wald Test of the Interaction			**0.039**			0.227			0.506
Religious Attendance		0.58	0.12	0.008	0.60	0.11	0.004	0.72	0.13	0.068
Sexual Identity										
	Heterosexual (Ref)									
	Bisexual	1.63	2.15	0.710	0.19	0.14	0.028	0.18	0.13	0.019
	Lesbian	1.37	1.82	0.815	0.23	0.17	0.042	0.23	0.17	0.047
Religious Attendance*Sexual Identity										
	Heterosexual (Ref)									
	Bisexual	1.89	0.51	0.018	1.48	0.32	0.066	1.17	0.25	0.482
	Mostly lesbian/Lesbian	1.53	0.42	0.122	1.56	0.32	0.028	1.27	0.27	0.268
	Wald Test of the Interaction			0.052			0.066			0.533

*Notes*. Survey weighted logistic regression models testing interactions adjusted for age, race/ethnicity, educational attainment, current employment, “partnered” relationship status, religious preference, and the sample from which the participant was recruited.

### 3.3 Models adjusting for religious environment

To test whether the significant interaction effects held after adjusting for religious environment, we reran the analyses testing the interactions of religiosity with sexual identity on AUD including the additional covariate. The differential effects of religious importance with AUD for sexual minorities relative to heterosexuals were attenuated and no longer statistically significant (tables available from corresponding author).

## 4 Discussion

In the current study we examined differences in the associations of religiosity and importance of spirituality with AUD and marijuana use by sexual identity in a large sample of adult sexual minority and heterosexual women. Consistent with prior research, importance of spirituality, importance of religion, and participation in religious services were independently associated with lower odds of substance use. However, this protective effect varied by sexual identity, particularly in regard to AUD.

In analyses of the interaction between spirituality and the study outcomes, we found that greater importance of spirituality was associated with greater odds of AUD among both lesbian and bisexual women, but was protective for heterosexual women. Among study participants who reported the highest levels religious importance odds of AUD were also greater among lesbian women than heterosexual women. These findings are consistent with minority stress theory, which suggests that religiosity and spirituality are less protective for SMW than heterosexual women and, in some cases, may contribute to greater risk of substance use. Findings are also consistent with research results indicating that religiosity is not protective against, and is sometimes associated with, increased heavy episodic drinking among adolescent and young adult SMW (Rostosky et al., 2008, Rostosky et al., 2014). Although our study did not explore participants’ subjective experiences and meanings of religiosity and spirituality, findings from qualitative research suggests that although religion or spirituality may provide support for some sexual minorities, it can also be associated with unique stressors (e.g., conflict, discrimination, rejection, and feelings of loss or alienation) which may contribute to adverse psychological and health outcomes (Wilkinson & Johnson, 2020). It may also be possible that some SMW are turning to accessible coping outlets to deal with minority stress—including both spirituality and substance use.

Findings from tests of interaction between religious attendance and sexual identity approached but did not reach statistical significance in relation to past year AUD or any marijuana use in the past year. Lack of significant differences may be related to the markedly lower levels of religious service attendance reported by SMW relative to heterosexual women, which may have reduced our ability to detect differential risk by sexual identity. These findings underscore the importance of future research considering religious behavior, such as attending religious services, in addition to religiosity or spirituality, given the potential of each to contribute to disparately negative behavioral health outcomes for SMW.

We also explored potential differences in outcomes among participants based on religious environment—specifically, whether the study outcomes differed for participants involved in religious environments that were unwelcoming to LGBT people. Differences by sexual identity in interaction models were attenuated and no longer significant when we added this variable. Our ability to explore this question in greater depth was limited by the relatively small number of SMW participants who reported that they attended services in unwelcoming religious environments (14 bisexual women [4.4 %] 12 lesbian women [2.8 %], and 226 [41.2 %] heterosexual women). Although the percentage of participants reporting attendance at LGBT welcoming environments was similar across sexual identity groups (between 11.8 % and 12.8 %), over 80 % of SMW, compared with 48.5 % of heterosexual women, described themselves as not affiliated with or attending services. These demographic differences are consistent with literature suggesting that sexual minorities are more likely than heterosexuals to dissociate from religious institutions entirely or seek alternatives to disaffirming religions (Scheitle and Wolf, 2017, Woodell and Schwadel, 2020). Studies with larger samples of SMW who attend religious services that are both welcoming and unwelcoming of LGBT people are needed to explore the potential impact of the immediate religious environment on substance use outcomes.

## 5 Limitations

Findings should be interpreted in the context of study limitations. Although the SMW participants were drawn from two large national panel samples of SMW, they were not recruited using probability sampling methods, which may limit generalizability. As noted above, the great majority of SMW did not participate in religious services, which limited our ability to explore the impact of religious environment on substance use outcomes. There were also some limitations related to measurement. We assessed importance of religion, religious attendance, and importance of spirituality each with a single item. Although the use of single items are common in survey research, there are other measures that capture different dimensions of religiosity not captured in the current study, such as organizational, nonorganizational, and subjective religiosity (Koenig & Büssing, 2010); daily spiritual experiences such as awe, inner peace, gratitude, transcendent experiences (Underwood & Teresi, 2002); or facets of religiosity that may be particularly salient to health such as religious coping (Boudreaux et al., 1995) and religious social support (Fiala et al., 2002). It is possible that a measure of religious coping or a multi-dimensional measure of spirituality would have yielded different results. Furthermore, it was not possible to assess the degree to which participants conflated religiosity and spirituality; multi-dimensional measures may have allowed for a more nuanced exploration of the impact of spirituality independent of religiosity. Measures of religious environment also differed between the SMW and heterosexual women, which may have contributed to the different distributions of “non-affirming” attendance by sexual identity. Given research suggesting differences in perceived importance of religion and religious affiliation by race and ethnicity among SMW (Barnes & Meyer, 2012; Drabble, Veldhuis, et al., 2018; Walker & Longmire-Avital, 2013), future studies might examine possible subgroup differences in the associations of religiosity and spirituality to substance use outcomes. Finally, differences between the two panel samples may have influenced the findings in the current study. Although research suggests that substance use is typically greater among SMW relative to heterosexual women regardless of the sample or measures used (Drabble, Trocki, et al., 2018; Karriker-Jaffe et al., 2022), LGBT specific panels may reach individuals whose characteristics differ (e.g., higher education, greater LGBT identity salience) than LGBT peers recruited from general samples (Karriker-Jaffe et al., 2022).

### 5.1 Conclusions

Findings from this study contribute to previous research suggesting that religiosity and spirituality are less protective against alcohol and marijuana use among SMW than among heterosexual women, and, in fact may be a risk factor for some SMW. Furthermore, risk and protection may differ for lesbian and bisexual women. Findings underscore the importance of research on risk factors for substance use among SMW that include distinct measures of religion and spirituality, and that disaggregate bisexual and lesbian subgroups in analyses.

## Declaration of Competing Interest

The authors declare that they have no known competing financial interests or personal relationships that could have appeared to influence the work reported in this paper.
